# The Expanding Role of Mitochondria, Autophagy and Lipophagy in Steroidogenesis

**DOI:** 10.3390/cells10081851

**Published:** 2021-07-22

**Authors:** Geetika Bassi, Simarjit Kaur Sidhu, Suresh Mishra

**Affiliations:** 1 Department of Physiology and Pathophysiology, Faculty of Health Sciences, University of Manitoba, Winnipeg, MB R3E 3P4, Canada; g1@myumanitoba.ca (G.B.); sidhus8@myumanitoba.ca (S.K.S.); 2 Department of Internal Medicine, Faculty of Health Sciences, University of Manitoba, Winnipeg, MB R3E 3P4, Canada

**Keywords:** adrenal gland, autophagy, cholesterol, lipophagy, mitochondrial, ovary, placenta, testis

## Abstract

The fundamental framework of steroidogenesis is similar across steroidogenic cells, especially in initial mitochondrial steps. For instance, the START domain containing protein-mediated cholesterol transport to the mitochondria, and its conversion to pregnenolone by the enzyme P450scc, is conserved across steroidogenic cells. The enzyme P450scc localizes to the inner mitochondrial membrane, which makes the mitochondria essential for steroidogenesis. Despite this commonality, mitochondrial structure, number, and dynamics vary substantially between different steroidogenic cell types, indicating implications beyond pregnenolone biosynthesis. This review aims to focus on the growing roles of mitochondria, autophagy and lipophagy in cholesterol uptake, trafficking and homeostasis in steroidogenic cells and consequently in steroidogenesis. We will focus on these aspects in the context of the physiological need for different steroid hormones and cell-intrinsic inherent features in different steroidogenic cell types beyond mitochondria as a mere site for the beginning of steroidogenesis. The overall goal is to provide an authentic and comprehensive review on the expanding role of steroidogenic cell-intrinsic processes in cholesterol homeostasis and steroidogenesis, and to bring attention to the scientific community working in this field on these promising advancements. Moreover, we will discuss a novel mitochondrial player, prohibitin, and its potential role in steroidogenic mitochondria and cells, and consequently, in steroidogenesis.

## 1. Introduction

Steroid hormones are an important class of regulatory molecules, which are synthesized mainly in the adrenal glands, the ovary, and the testis, in response to steroidogenic stimuli, and regulate growth and drive a variety of physiological processes, such as reproduction and metabolism [1]. The importance of steroid hormones are evident from their wide-ranging essential functions in the body physiology, including carbohydrate metabolism, stress response, and in the regulation of salt balance pertaining to the maintenance of blood pressure by adrenal corticoids to the role of sex steroid hormones in males and females in the development of secondary sex characteristics, maintenance of reproductive functions, and perpetuation of life, as well as an essential role of progesterone for a successful pregnancy [2]. The steroid hormones can be distinguished from one another by their diverse physiological actions in the body; however, an overarching commonality among them is that they all are produced from cholesterol. Thus, an advanced understanding of steroid hormone biology is a requisite in the biomedical field.

**Figure 1:**
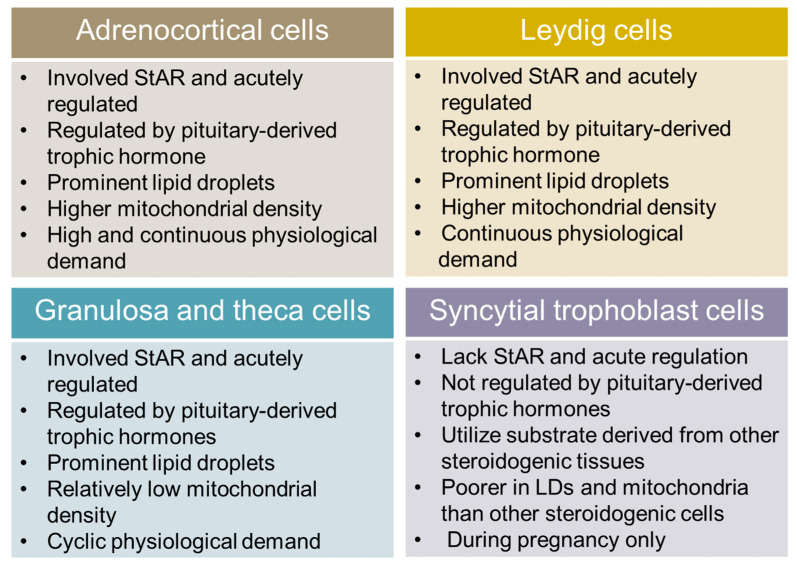
Similarities and differences between major steroidogenic cells in relation to physiological demand and cellular structure pertaining to lipid droplets and mitochondrial structure/content.

The first step in the biosynthesis of steroid hormones is the enzymatic cleavage of a six-carbon unit side chain of cholesterol molecule by the 20–22 desmolase/lyase activity of the cytochrome P450 side chain cleavage (P450scc) enzyme system located in the inner mitochondrial membrane (IMM) [3,4]. Steroidogenesis is a finely compartmentalized, multistep enzymatic process in steroidogenic cells, which involve different cellular compartments, including the cytoplasm, mitochondria, and the smooth endoplasmic reticulum (SER). The initiation of steroidogenesis that involves the enzymatic cleavage of the cholesterol side chain is conserved across steroidogenic cells. The enzyme P450scc localizes to the matrix side of the IMM [3,4], which makes the mitochondria central to steroidogenesis. Thus, it is not surprising that steroidogenic cells (e.g., adrenocortical cells in the adrenal glands, the granulosa, and theca cells in the ovary, Leydig cells in the testis, and syncytial trophoblast cells in placenta) are rich in mitochondria [5,6,7]. In this review article, we will refer them as ‘steroidogenic mitochondria’ because of their inherent ability to initiate steroidogenesis not shared by the mitochondria from non-steroidogenic cells. For instance, steroidogenic acute regulatory protein (StAR)-mediated cholesterol transport to the mitochondria and its subsequent utilization by the enzyme P450scc. As mitochondria is an important signaling hub, it is likely that this special attribute of steroidogenic mitochondria might drive many aspects of steroidogenesis in a steroidogenic cell type-specific manner because the physiological demand for each steroid hormone varies substantially. Such a difference in steroid hormone levels in the body’s physiology may explain why the structure, number and distribution of mitochondria vary substantially across steroidogenic cells (Figure 1).

For example, mitochondrial features and the distribution in adrenocortical cells and Leydig cells are relatively more prominent in comparison to ovarian and placental steroidogenic cells [8]. While such differences in mitochondria in steroidogenic cells may be explained on the basis of their steroidogenic capacity and the physiological need of different steroid hormones, which ranges from picomoles to micromoles (Figure 1), it is also possible that a substantial difference in mitochondrial attributes in different steroidogenic cells are a reflection of their need to maintain the cholesterol homeostasis required to maintain basal, acute, and chronic steroidogenesis in a steroidogenic cell type-specific manner.

**Figure 2:**
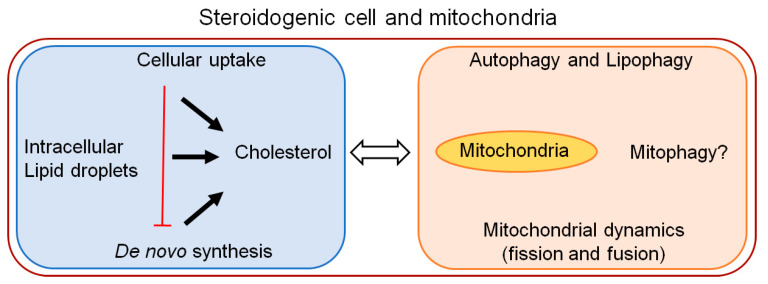
Schematic diagram depicting known and potential relationship between cholesterol homeostasis and mitochondrial attributes in major steroidogenic cells. The interplay between different intrinsic factor expected to vary under basal, acute and chronic steroidogenic state (as applicable) because of a wide range of different steroid hormone levels and their physiological need.

The steroid hormones are not stored in secretory vesicles like peptide hormones, but released into the blood upon their biosynthesis [8]. This instant set-up between the biosynthesis and release of steroid hormones is expected to require an arrangement to maintain the readily available cholesterol pool within steroidogenic cells, because the cholesterol contents of mitochondrial membranes, especially the IMM, where steroidogenesis begins, is insufficient to support steroidogenesis [8]. Emerging evidence suggest that mitochondrial dynamics, autophagy, and related lipophagy play crucial roles in intracellular cholesterol uptake and in the maintenance of cholesterol homeostasis in steroidogenic cells, and consequently support steroid hormone production to maintain physiological functions (Figure 2).

Thus, an expanding role of cell-intrinsic processes (e.g., autophagy, lipophagy and mitochondrial attributes) in steroidogenesis have created a need for a timely review article for the benefit of the scientific community engaged in this field, and to facilitate research to answer fundamental and emerging unanswered questions. In this review, we will focus on the cell-intrinsic attributes of different steroidogenic cells involved in cholesterol handling and mitochondrial activities. We have two interconnected goals: first, to discuss the importance of various processes involved to maintain a readily available pool of cholesterol for the varying need of steroidogenic demands, and second, to review the growing role of mitochondria, autophagy, and lipophagy, and other related activities in a steroidogenic cell type-specific manner to meet the diverse physiological demand of each steroid hormone (Figure 2). While several excellent review articles are available on receptor mediated cholesterol uptake (e.g., SR-B1 and LDL receptors), cholesterol mobilization and transport to mitochondria in steroidogenic cells [2,3,4,5,6,7,8], to the best our knowledge there is a lack of review articles in current literature, which provide a broader perspective in this context integrating the accruing role of autophagy, lipophagy and mitochondrial attributes in relation to steroidogenesis, which is the focus of this review. In addition, we will discuss a novel mitochondrial player, prohibitin (PHB), and its potential role in integrating steroidogenic mitochondria with cholesterol handling and steroidogenesis in a context-dependent manner based on our current knowledge of PHB and the PHB family of proteins in mitochondrial biology and lipid metabolism. In this review, we will not discuss signaling and cellular events in cholesterol sequestering and trafficking in steroidogenic cells but instead will focus on accruing evidence related to autophagy, lipophagy, and the mitochondrial dynamics involved in handling the cholesterol pool in the cytoplasm of steroidogenic cells, and highlight any pertinent questions that may arise in this exploration.

## 2. Steroidogenic Cells and Steroidogenic Mitochondria

A cell is classified as “steroidogenic” if it expresses the enzyme P450scc and therefore can catalyze the first reaction of steroidogenesis (i.e., the conversion of cholesterol to pregnenolone). Many cells can transform steroids produced in other cells (e.g., adipocytes), but only cells expressing P450scc are steroidogenic [2]. As P450scc resides in the IMM, the mitochondria of steroidogenic cells can be called steroidogenic mitochondria by the same token, because of their distinct ability to begin steroidogenesis (as mentioned earlier), which is not shared by the mitochondria from non-steroidogenic cells. However, as IMM is cholesterol-poor, there is a prerequisite for steroidogenesis by P450scc, i.e., for a cholesterol import to the mitochondria, which is mediated through the START domain containing protein (e.g., StAR in steroidogenic adrenocortical and gonadal cells and metastatic lymph node 64 (MLN64) protein in placental cells [2]. Thus, P450scc is essential, but not sufficient, to initiate steroidogenesis by itself because of its physical location in the IMM and is assisted by proteins involved in cholesterol transport to the mitochondria. Consequently, unlike many metabolic pathways (e.g., glycolysis, citric acid cycle, fatty acid synthesis), the committed step in steroidogenesis is not an enzymatic reaction (i.e., the P450scc-mediated conversion of cholesterol to pregnenolone, which was previously thought to be one), but rather, cholesterol transport to the mitochondria that is mediated by the StAR protein [2]. In addition to P450scc, 11-hydroxysteroid dehydrogenase and aldosterone synthase in adrenocortical cells localize to the mitochondrial IMM, and 3β-hydroxysteroid dehydrogenase has been reported to be present in the mitochondria [9]. In fact, 3β-HSD was first isolated from the mitochondria [10]. Furthermore, the mitochondria in steroidogenic cells are responsive to the actions of trophic hormones, and steroidogenic cells have evolved a variety of ways to acquire and mobilize cholesterol for the maintenance of basal, acute, and chronic steroidogenesis. Thus, it is a combination of interconnected features spanning different cellular compartments, which define the cell type-specific function of a steroidogenic cell and the steroidogenic mitochondria (Figure 2).

The precursor cholesterol for steroidogenesis is known to come from at least three sources, such as the mobilization of cholesterol from the lipid droplets (LDs), the uptake of circulating cholesterol esters, and the de novo synthesis of cholesterol, which have been described extensively in many review articles [3,11]. However, our understanding of the relative contributions of various sources of cholesterol to the different stages of steroidogenesis (i.e., basal, acute, and chronic) in major steroidogenic cells remains limited. It is likely that these processes work in a coordinated manner to maintain the physiological needs of different steroid hormones (which vary substantially) in a context-dependent manner. For example, the mobilization of cholesterol from LDs may play a major role in the acute response to trophic hormones, whereas the de novo synthesis of cholesterol may be a major contributor for basal and chronic steroidogenesis, and in the cellular uptake of cholesterol in replenishing the depleted, readily available pool due to the acute response, and in the maintenance of a chronic response. Similarly, the instantaneous contribution of cellular uptake and the de novo synthesis of cholesterol for acute steroid production in response to trophic hormones is expected to be minimal (Figure 2).

In addition to steroidogenic cells, the scavenger receptor class B type I (SR-BI) that are involved in cellular cholesterol uptake are found in many non-steroidogenic cell types, such as macrophages and endothelial cells [12]. In testicular interstitium, the predominant cell types are Leydig cells and macrophages, and both cell types appear to be dependent on each other. A decrease in the number of one cell type (by genetic or pharmacological approaches) leads to a corresponding decrease in other cell types and vice versa [3,11]. In addition, testicular macrophages have been implicated in supporting Leydig cell steroidogenesis, especially in bypassing the StAR-mediated cholesterol transport by providing 25-hydroxysteroid cholesterol to the Leydig cells [13]. In addition, the potential role of phagocytic activities of macrophages in cholesterol recycling in testicular interstitium (and potentially in other steroidogenic tissues, such as adrenals and ovaries) may not be discounted.

### 2.1. Steroidogenesis and Mitochondrial Structures—The Role of Steroidogenic Enzymes

Structural changes in the mitochondria appears to be an integral feature of the differentiation of steroidogenic cells, which involve an acquisition of steroidogenic capability due to the expression of steroidogenic enzymes during development [14,15]. For instance, the differentiation of non-steroidogenic cytotrophoblasts into steroidogenic syncytial trophoblasts during the development of placenta involves increased expression of P450scc, which coincide with structural changes in the mitochondria. This includes a reduction in mitochondrial size and a change in the shape of mitochondrial cristae [16]. A difference in mitochondrial cristae shape have also been reported between non-steroidogenic cells of adrenal medulla and steroidogenic adrenocortical cells [17]. Interestingly, the mitochondrial cristae structure and inter-cristal space also differ between different steroidogenic cells of the adrenal cortex [14,15]. For example, mitochondrial cristae are lamellar with a wide inter-cristal space in aldosterone producing zona glomerulosa, whereas they are vesicular in cortisol/corticosterone producing the zona fasciculata [15]. Moreover, the different steroidogenic cells of the ovary also display differences in their mitochondrial cristae structures. In granulosa cells, the mitochondria have an elongated shape with lamellar cristae, whereas in luteal cells, the mitochondria are spherical in shape with tubular-vesicular cristae [15]. As the steroidogenic capacity (and corresponding mitochondrial attributes) of different steroidogenic cells vary substantially because of their diverse physiological need, their relationship with mitochondrial shape and cristae structure would imply that steroidogenic enzymes in the mitochondria may have a role in controlling mitochondrial structure and function. The findings from transgenic mice that overexpress Cyp11a1, as well as *StAR* and *Cyp11A1a* knockout mouse models are consistent with this notion. For instance, the mitochondria of the luteal cells of Cyp11A1 transgenic mouse model are elongated from their normal spherical shape [15]. Furthermore, the impact of *Cyp11A1* and *StAR* knockout on the mitochondria of zona fasciculata cells differ from each other, which is more severe in the former than the later [18,19]. As Cyp11a1 and StAR work tandemly in the beginning of steroidogenesis and their knockout models display overlapping phenotypes, their differential effect on mitochondrial phenotype in steroidogenic cells are likely due to the direct effect of gene deficiency rather than secondary to dysregulation of cholesterol handing. In this context, it is important to note that placental and ovarian steroidogenic cells undergo proliferation and differentiation in each cycle, which is not the case with the adrenals and testis; thus, the workload and related mitochondrial attributes are expected to vary substantially to meet their respective physiological demands.

### 2.2. Hormone-Induced and Cell-Intrinsic Processes in Steroidogenic Cells

Tropic hormone-induced steroidogenesis in the adrenals and the gonads have been studied extensively. However, the context-dependent potential contributions of cell-intrinsic events remain largely unexplored. New evidence demonstrating the importance of autophagy and lipophagy in steroidogenesis in combination with our previous knowledge of cholesterol homeostasis and mitochondrial dynamics in steroidogenic cells have created an opportunity to explore these aspects and attain new insights. For example, preclinical models mimicking congenital adrenal hyperplasia (CAH) phenotype provides an excellent example to investigate cell-intrinsic processes and the physiological needs of cell type-specific steroidogenesis on the varying impacts of the loss-of-function mutation in StAR.

The characteristic features of lipoid CAH are hypertrophied adrenals with enlarged lipid droplets because of high ACTH level and renin activity, and substantially reduced serum levels of steroid hormones [20]. Bose et al. [21] proposed a two-hit model to explain the pathophysiology of lipoid CAH. According to this model, the loss of StAR activity due to naturally occurring mutation is the first hit, causing reduced steroidogenesis and consequently an increase in trophic hormones (i.e., ACTH, LH and FSH) [22]. Higher trophic hormones lead to increased production of secondary messenger and consequently increased cholesterol uptake and biosynthesis by adrenocortical cells. This imbalance in cholesterol accumulation and utilization leads to the second hit—mitochondrial damage due to lipotoxicity and the loss of residual steroidogenic capacity [21], which explains the phenotypic manifestation in different steroidogenic cell types/tissues. Thus, a balance between the cholesterol availability and steroidogenesis is critical for the normal functioning of steroidogenic cells. For instance, earlier manifestation of the loss of StAR function in steroidogenic cells of the fetal testes and after birth in adrenocortical cells correlates with the initiation of steroidogenesis in both tissues, respectively [22]. On the other hand, the fetal ovary does not make steroids and remains unstimulated and therefore remains normal until puberty. In aggregate, this evidence suggests that a coordination between the upstream stimuli and downstream functions are important to maintaining mitochondrial functions in steroidogenic cells. Of note, the StAR-knockout mouse model recapitulates the phenotype of StAR deficiency in humans. Thus, the phenotypic manifestation of StAR deficiency precisely correlates with the sensitivity of steroidogenic cells to respective tropic hormones and their steroidogenic activity during different stages in life. In summary, there is much to learn from the comparative accounts of steroidogenesis in different steroidogenic cells.

### 2.3. The Importance of Mitochondrial Dynamics in Steroidogenesis

The initiation of the biosynthesis of steroid hormones occurs in the mitochondria, which are known to undergo dynamic changes called mitochondrial fission and fusion [23]. Therefore, it is likely that the cellular and molecular changes in mitochondria would influence steroidogenesis. Indeed, many studies have shown that mitochondrial dynamic changes are closely associated with the biosynthesis of steroid hormones in steroidogenic cells [5,6,7,8]. For example, cAMP-induced steroid hormone production has been reported to be accompanied by increased mitochondrial mass [23], specifically an increase in mitochondrial fusion, whereas a reduction occurs in mitochondrial fission. Among the mitochondrial proteins that are involved in shaping the mitochondria, dynamin-associated protein 1 (Drp1) level was found altered in response to db-cAMP stimulation. Particularly, an increase in the phosphorylation of Drp1 at Ser637 correlated with steroid hormone production in the primary adult rat Leydig cells and in a model murine cell line of Leydig cells. In addition, gonadotropin administration was found to alter the status of Drp1 phosphorylation in the Leydig cells isolated from immature rat testes [23]. Overall, mitochondrial dynamics at large were found to be directly linked to steroidogenesis, and Drp1 was found to play an important regulatory role during steroidogenesis [23]. Thus, cAMP-PKA pathway, which plays a central role in the Leydig cell steroidogenesis is also involved in the regulation of mitochondrial dynamics to facilitate steroidogenesis. Moreover, hypogonadism was found to affect mitofusin (Mfn1) and mitofusin (Mfn2) in the Leydig cells by reducing the transcription of Drp1, as well as Mfn1 and Mfn2, without changing protein optic atrophy 1 (Opa1) levels [23].

The differences between placental trophoblasts and syncytiotrophoblasts that sustain progesterone production during human pregnancy is accompanied by mitochondrial fragmentation and cristae remodeling [24]. Subsequent work revealed that the mitochondria-shaping Opa1 controls the efficiency of steroidogenesis in BeWo cells [24]. This finding further supports the notion that structural changes in mitochondria play a role in steroidogenesis. However, a similar change in mitochondrial structure has not been reported in other steroidogenic cell types, which express StAR for cholesterol transport to the mitochondria. Thus, there appears to be a link between steroidogenesis and mitochondrial structure.

### 2.4. Steroidogenic Mitochondria—A Comparative Account

While the final steroid hormone product differs in different steroidogenic cell types, the first step in the steroidogenic pathway is precisely similar, which is catalyzed by the enzyme P450scc located in the IMM. In addition to the common first step of the steroidogenic pathway, the final steps in the biosynthesis of glucocorticoids and mineralocorticoids are also catalyzed by two closely related mitochondrial enzymes: CYP11B1 and CYP11B2 (11β-hydroxylase and aldosterone synthase), located in the IMM [25]. Both enzymes display differential expression in three different steroidogenic cell types in the adrenal cortex, which in turn contribute to cell type-specific corticosteroid production. Moreover, 3β-hydroxysteroid dehydrogenase has also been reported to localize to the mitochondria [22]. In this context, it is important to note that three different steroidogenic cell types of the adrenal cortex, comprising of three different zones (i.e., the glomerulosa, fasciculata and reticularis) display morphological differences in photomicrographs, which are easily distinguishable from each other. However, it remains unclear what contributes to such morphological differences, despite their common functions (i.e., the production of the steroid hormone). It is likely that the physiological demand of the steroid hormone they produce, and the corresponding requirements of the steroidogenic machinery (e.g., the expression levels of steroidogenic enzymes involved, mitochondrial numbers, and the readily available cholesterol pool) contribute to such differences. It has been suggested that mitochondrial steroidogenic enzymes play a role in the regulation of mitochondrial morphology and structure, which in turn influence steroid production [26]. For example, mitochondrial cristae in steroidogenic cells are vesicular and/or tubular in shape, which relate to the degree of their steroidogenic function [26].

Steroidogenic mitochondria, particularly in adrenal and gonadal steroidogenic cells, are affected differently by the actions of the pituitary tropic hormones during the acute and the chronic response [27]. The acute response begins in minutes after the binding of the pituitary tropic hormone to their cognitive receptors. This is accomplished by increased trafficking of cholesterol from the cytoplasmic compartment to P450scc in the IMM and does not involve change in the levels of proteins involved in catalyzing this enzymatic step [27], whereas the longer time effects involve upregulation of the protein levels of steroidogenic enzymes. Thus, the control of steroidogenesis by the mitochondrion itself is exerted at two levels: first, the regulation of cholesterol pool as a precursor substrate, and second, the regulation of the mitochondrial import and processing of the nuclear transcribed steroidogenic enzymes, including 11β-dehydrogenase and aldosterone synthase for adrenal steroidogenesis, which may vary under basal and stimulated states. An important point that needs to be considered in this context is the potential consequences of the NADPH-utilizing metabolic reactions on portioning of NADH for the respiratory chain and on the proton gradient, which is expected to vary between the adrenocortical and other steroidogenic cells. This is because of differences in the number of steroidogenic enzymes present in the IMM in adrenocortical cells and other steroidogenic cells, and differences in the physiological levels of the respective steroid hormones produced. These differences are expected to create mitochondrial heterogeneity between steroidogenic cells, which is a topic that currently remains unclear to us. Furthermore, the mitochondria in steroidogenic cells not only have enzymes for steroidogenesis, but also have unique mechanisms for regulating cholesterol availability of these enzymes—regulation of StAR levels and cholesterol trafficking.

Structural changes in steroidogenic mitochondria have been reported during the development and differentiation of steroidogenic cells. For instance, the differentiation of cytotrophoblasts into syncytial trophoblasts is marked by a substantial increase in the expression levels of CYP11A1 and vesicular cristae [16]. As discussed in the Section 2.1, a correlation between mitochondrial structure and steroidogenesis has also been reported in adrenocortical cells and ovarian granulosa and theca cells [14,15]. It is possible that steroidogenic enzymes in the mitochondria play a role in regulating mitochondrial attributes in steroidogenic cells [26]. Thus, the relationship of mitochondrial structure and steroidogenesis appears to be two-ways in steroidogenic cells, as hormone-induced steroidogenesis leads to changes in mitochondrial structure and dynamics, whereas manipulation of the mitochondrial remodeling affect steroidogenesis [26,28], which in turn feedback to steroidogenic stimuli and creates a regulatory cycle. For example, when the level of OPA1 in BeWo cells (a model human placental cell line) is reduced, cholesterol flux into mitochondria and steroid production are increased [28]. Thus, there are two specific aspects of the mitochondria in steroidogenic tissues, including mechanisms to deliver cholesterol to the mitochondria for steroidogenesis, and mitochondrial enzymes in the inner mitochondrial membrane for the initiation of steroidogenesis [22].

In addition to the P45scc that is present in all steroidogenic cells, adrenocortical mitochondria contain two additional P450 enzymes: 11β–hydroxylase in zona fasciculata and aldosterone synthase in zona glomerulosa, which also localize to the IMM. The former catalyzes the conversion of 11-deoxycortisol to cortisol, while the latter catalyzes the conversion of deoxycorticosterone to aldosterone [29,30,31]. All three steroidogenic enzymes that are localized to the IMM use reducing equivalents provided via ferredoxin reductase and ferredoxin. Thus, a demand for reducing equivalents in adrenocortical cells is expected to be much higher than other steroidogenic cell types in the gonads and their high demand in adrenocortical cells may compete with non-steroidogenic processes that use them. It is likely that these differences in the needs of different steroidogenic cells would affect mitochondrial attributes differently and may contribute to differences in mitochondrial structure, number and function in different steroidogenic cell types and tissues.

## 3. Placental Steroidogenesis—What We Can Learn from the Similarities and Differences with Adrenal and Gonadal Steroidogenesis?

Despite several commonalities with adrenal and gonadal steroidogenesis (e.g., ability to synthesize steroid hormones by specific cell types), placental steroidogenesis displays certain unique arrangements and characteristics, which are shared among placentae from different species. These include differences in regulatory mechanisms that control the expression of steroidogenic enzyme genes from other steroidogenic tissues, an interplay between maternal and fetal compartments to support placental steroidogenesis, and the regulatory influences on maternal hypothalamic-pituitary-gonadal axis and fetal adrenal corticosteroids [32]. Importantly, the ability to metabolize steroid hormones derived from the maternal ovary and fetal adrenal is one common feature of trophoblast cells, despite the marked differences in placental morphologies in different species. Thus, the cell-intrinsic steroidogenic characteristics of trophoblasts, particularly cholesterol handling and mitochondrial attributes, are expected to vary substantially from those of the ovarian granulosa and theca cells, as well as testicular Leydig cells. A fitting example of this is the lack of StAR expression and acute steroidogenesis in the placenta, which are characteristic features of adrenocortical and gonadal steroidogenesis. Due to this fundamental difference between the two steroidogenesis types, it is likely that the mechanisms involved in cholesterol handing and mitochondrial activities between them will also vary substantially. For example, the regulatory mechanisms involved in controlling the expression of the placental P450 gene are different than in the adrenal cortex and gonads. In addition, it appears that that the second messenger cAMP, which plays a central role in adrenal and gonadal steroidogenesis, does not have a prominent role in regulating placental steroidogenesis in many species [32]. Moreover, the trophoblast 3β-HSD is different from that which is expressed in the gonads and adrenal cortex, and the placental aromatase gene is transcribed from unique promoters [32]. Furthermore, unlike adrenals and gonads, which synthesize steroid hormones from the precursor cholesterol molecule, the placenta is able to utilize steroid precursors contributed by both the mother and the fetus, and are influenced by their hormones. Substantial evidence exists in literature to suggest that the placenta engages in a dynamic steroid-mediated dialogue with both the maternal pituitary and ovary and the fetal adrenal cortex [32]. The unique aspects of placental steroidogenesis mentioned above raise many important biological questions with unclear answers (see Appendix A. Outstanding Questions).

Like the adrenal cortex and the gonads, progesterone production by the human placenta requires pregnenolone synthesis from cholesterol by cytochrome P450scc [33,34,35,36]. Most steroidogenic tissues use StAR protein to deliver cholesterol to the IMM where P450scc resides. However, instead of StAR, the human placenta express MLN64 (metastatic lymph node 64 protein), which has a C-terminal domain homologous to StAR [33,34]. Many studies have shown that the cholesterol binding domain of both proteins in human have similar biophysical and functional properties and are able to support steroidogenesis in placental tissue and their cell derivatives in different species [37,38,39,40,41,42,43,44,45,46,47,48].

A unique aspect of placental steroidogenesis is its close relationship with maternal and fetal steroidogenesis. For example, E2 and P4 produced from maternal ovaries play important roles in placenta formation and function during early stages of pregnancy. Subsequently during later stages, the placenta itself acquires the ability to produce the P4 required to maintain pregnancy [49]. In this context, it is important to note that while the E2 and P4 produced from the maternal ovaries are under the control of maternal pituitary gonadotropins, this is not the case with placental P4 production. In addition, the placenta also utilizes steroidogenic metabolites of the fetal adrenal glands [49]. It is likely that because of this arrangement, placental steroidogenesis would not require intracellular arrangements and regulatory mechanisms (or would substantially vary from) that are required for steroidogenesis in other steroidogenic tissues (i.e., adrenal, ovary and testis).

Pregnancy in women is marked by substantial changes in their endocrine system [50]. For instance, levels of E2 and P4 dramatically increase during pregnancy, suppressing the hypothalamic-pituitary axis and subsequently the ovarian and menstrual cycle [51]. Such a shift in the production of steroid hormones would require an enhanced expression and activity of the steroidogenic enzymes in the placental tissue, resulting in increased serum and placenta levels of E2 and DHEA near the end of gestation.

Many placental species, including human, does not express 17α-hydroxylase [39,52,53]. Therefore, placental estrogen synthesis in them depends on a source of androgen precursors from the fetus steroidogenic tissues (e.g., the fetal adrenal glands and the gonads). However, the trophoblast cells in some species (e.g., rat, pig, sheep, and cow) express 17α-hydroxylase [37,54,55,56,57,58] and can synthesize androgens. Thus, the expression of StAR gene appears to be limited to steroidogenic tissues, which exhibit acute regulation of steroidogenesis, but not in the placenta or placenta derived cells [59] suggesting involvement of other factors in cholesterol delivery to P450 system. Of note, the ability of N-218 MLN64 to transport cholesterol between the membranes of artificial phospholipid vesicles indicates that no other proteins are necessary for the transport activity of N-218 MLN64.

## 4. Cholesterol—Its Importance as a Starting Substrate and Need for Cholesterol Import to Mitochondria

Cholesterol, which is an essential component of all animal cell membranes, plays a critical role in defining membrane’s biochemical and biophysical characteristics [60]. Notably, the cholesterol content of the plasma membrane and different subcellular organelle membranes differ substantially. For example, relative to the ER and mitochondria membranes, the cholesterol content of the plasma membrane is approximately 40-fold higher [61]. As cholesterol levels are low in the mitochondrial membranes, cholesterol must be transported to the IMM for the initiation of steroidogenesis. In addition, the OMM and IMM must be supplied with cholesterol for the membrane’s biochemical and biophysical characteristics at large. Thus, the mitochondrial membranes in steroidogenic cells are distinct from non-steroidogenic cell types in relation to their dual requirements of cholesterol. First, the very low levels of cholesterol in the IMM allow to control steroidogenesis through the regulation of cholesterol transport to the IMM (including the acute regulation in response to steroidogenic stimulation), which serves as the ‘committed step’ in steroidogenesis and are distinct from the committed steps in other biosynthetic and metabolic pathways that are generally governed by specific enzymes. Second, because of the very low levels of cholesterol contents of the mitochondrial membranes, even small changes can have a substantial impact on the biophysical and functional characteristics of the membrane and likely to alert the mitochondria to changes in cholesterol content. Consistent with this notion, many studies have suggested that mitochondrial membrane cholesterol can influence mitochondrial function (independent of steroidogenesis) and may contribute to the pathology of diseases related with mitochondrial abnormalities. However, our current understanding of the mechanisms involved remain unclear. As steroidogenesis is essential to life, it is not surprising that many pathways for cholesterol supply to the steroidogenic mitochondria have been described. Thus, deficiency in one pathway can be taken care of by other pathways to ensure normal steroidogenesis. However, our current understanding of cholesterol delivery from the OMM to the IMM in general and its distribution between them when cholesterol levels change substantially in particular are largely unknown. This scenario raises an obvious question about steroidogenic cells because in these cells, cholesterol serves as the substrate for the synthesis of all steroid hormones [62]. To overcome such a challenging scenario, the steroidogenic cells have become highly evolved to perform cholesterol uptake, mobilization, and trafficking to the mitochondria to perform steroidogenic functions.

Cholesterol has many versatile characteristics. On the one hand, cholesterol plays a critical role in determining the biochemical and biophysical properties of cellular membranes, whereas on the other hand, cleavage of its six-carbon unit side chain from cholesterol molecule (at the beginning of enzymatic step in the steroidogenic pathway) in combination with its oxygenation at certain residues changes its chemical properties and functions [63]. This change in chemical properties dramatically shifts its biological role from membrane structure-function to cell signaling and transcriptional regulation [63].

Cholesterol may be produced de novo from acetate in steroidogenic cells through a series of enzymatic pathways mainly located in the endoplasmic reticulum (ER) [64]. However, circulating lipoproteins serve as a major source for most steroidogenic cholesterol. High-density lipoproteins (HDLs) and low-density lipoproteins are taken up via the scavenger receptor B1 (SR-B1) and by receptor-mediated endocytosis, respectively [22]. In addition, current literature suggests that steroidogenic cell-extrinsic (HDL- and LDL-mediated) and cell-intrinsic (de novo) supply of cholesterol works in a coordinated manner and are regulated by the intracellular pool of cholesterol [22]. Moreover, as the enzyme P450scc system, which is required for the initiation of steroidogenesis is localized to the IMM, the transfer of cholesterol from the OMM to the IMM is an essential step for steroidogenesis. Thus, the maintenance of the readily available intracellular cholesterol pool and its transport to steroidogenic mitochondria is central to steroidogenesis.

Cholesterol functions as the precursor substrate for the biosynthesis of all steroid hormones, which begins in the IMM by the cytochrome P450scc enzyme system leading to the formation of pregnenolone, the first steroid in the steroidogenic pathway. To ensure that this essential step proceeds normally, the steroidogenic cells have evolved a robust mechanism to maintain cholesterol pool in the cytoplasm and then traffic to the steroidogenic mitochondria when needed [62]. This is accomplished through a series of coordinated steps that involve various intracellular organelles, including lysosomes and lipid droplets for cholesterol mobilization, and the StAR protein for cholesterol trafficking to the steroidogenic mitochondria [3,11]. Of note, cholesterol content of the IMM is relatively very low in comparison with that of the OMM, and in steroidogenic cells, the pool of cholesterol available for steroidogenesis in the form of lipid droplets is segregated from the mitochondrial membrane cholesterol. Understanding the mechanisms that control mitochondrial cholesterol homeostasis and trafficking pertaining to steroidogenic and non-steroidogenic functions of mitochondria may provide new insights into diseases related to mitochondrial dysfunction.

### Heterogeneity in Cholesterol Distribution and Cellular Compartmentalization of Steroidogenesis

Cholesterol is a versatile lipid, which is synthesized by animal cells and is an integral component of their subcellular membranes [60]. Moreover, cholesterol is the precursor substrate for all steroid hormones. Cholesterol constitutes approximately 30–40% of total cellular lipids, and nearly 60–80% of total cellular cholesterol is present in the plasma membranes [60]. In addition to the plasma membranes, cholesterol concentrations are high in the Golgi apparatus. In contrast, cholesterol concentrations are very poor in the endoplasmic reticulum and mitochondrial membranes. Thus, cholesterol concentration is highly heterogeneous in cellular membranes, ranging from 0.5–1% in the SER to 50–60% in the plasma membranes, and 0.1% to 0.2% in the IMM and OMM, respectively [60]. In cellular membranes, cholesterol influences many biophysical and biochemical aspects of membranes and membrane protein functions. Such heterogeneity in cholesterol composition in different subcellular membranes (e.g., ER and PM) and within organelle membranes (e.g., IMM and OMM) would imply that cholesterol distribution and homeostasis must be carefully regulated in relation to membrane-specific functions. As IMM, where steroidogenesis begins, is cholesterol-poor in comparison to other subcellular membranes and compartments, it is predictable that the delivery of cholesterol to the IMM may serve as a critical regulatory step for steroidogenesis. As expected, many studies have established that this is indeed the case. Moreover, the low basal levels of cholesterol in mitochondrial membranes make the mitochondria sensitive to changes in cholesterol content, which can have a substantial impact on various non-steroidogenic functions of the mitochondria, as IMM is considered the most protein-enriched membrane in the cell. This would require a strict regulation of cholesterol handling in steroidogenic cells to protect mitochondria and mitochondria-mediated non-steroidogenic vital cellular functions. Thus, it is possible that the growing number of mitochondrial attributes and other cellular events (i.e., autophagy, lipophagy and mitochondrial dynamics) that are linked with cholesterol mobilization and trafficking in steroidogenic cells provide some flexibility to carry on steroidogenic and non-steroidogenic functions successfully under varying conditions and meet the diverse physiological needs of different steroid hormones. Thus, a possibility exists that autophagy, lipophagy and mitochondrial dynamics that have been implicated in supporting steroidogenesis protect the steroidogenic mitochondria from the potentially damaging effects of cholesterol, remove damaged mitochondria, and recycle biomolecules, including the maintenance of cholesterol homeostasis. It is likely that blocking or interfering with these protective mechanisms would lead to mitochondrial dysregulation and impaired steroidogenesis. Thus, an execution of the cell type-specific function of mitochondria (e.g., steroidogenesis) would necessitate a protection of the cell-neutral functions of mitochondria in an integrated fashion. Consistent with this notion, it is important to note that these activities (i.e., autophagy, lipophagy, mitochondrial dynamics), which have been implicated in steroidogenesis, are also operative in non-steroidogenic cells with cell type-specific mitochondrial functions, such as brown adipocytes, cardiomyocytes, hepatocytes. In addition, the compartmentalization of intracellular cholesterol in lipid droplets (LDs) in steroidogenic cells may not only provide a readily available pool for steroidogenesis, but also allow its portioning from the structural membrane cholesterol, and in protecting the functional integrity of mitochondrial and other organelles. Notably, once cholesterol is imported into the mitochondria, the steroidogenic process proceeds uninterrupted. Thus, the mitochondria may be seen as a portioning point between the processes involved in maintaining the readily available pool of precursor substrate cholesterol and the multi-enzymatic steps of steroidogenesis in the IMM and SER. This arrangement makes sense and may have evolved to protect both cell-specific and cell-neutral mitochondrial functions from the potential disruptive effects of high cholesterol levels in steroidogenic cells. For example, increased mitochondrial cholesterol levels have been reported to decrease membrane fluidity [65,66,67,68,69], which can affect the function of mitochondrial membrane proteins [70], including different transporters for metabolites [71,72,73,74]. In addition, functional changes associated with increased mitochondrial cholesterol are similar to the effect of lipotoxicity in many cell types, including increased reactive oxygen species production and a pro-oxidative environment [75,76,77,78,79,80], increased opposition to mitochondrial membrane permeabilization, and decreased oxidative phosphorylation [65,67,81,82]. Thus, steroidogenesis involved a coordination between different cellular compartments. As such, steroidogenesis may be conceptualized as a compartmentalized process that involves a fine coordination of events in the different cellular compartments (e.g., the cytoplasm, mitochondria, and SER) to operate the cell-specific needs of the steroidogenic mitochondria without affecting the mitochondria’s cell-neutral global functions.

## 5. Autophagy and Lipophagy in Cholesterol Homeostasis and Steroidogenesis

Autophagy is an evolutionarily conserved, cellular degradative pathway that involves the systematic degradation of select cytoplasmic components and organelles [82]. During this process, the cellular material destined for degradation is captured by the autophagosome, a double phospholipid bilayer organelle, which then fuses with the lysosome to degrade internalized debris to recycled cellular materials to maintain cell homeostasis [83,84]. Subsequent studies revealed that autophagy is essential for cell survival, differentiation, and homeostasis, and plays an important role during development and its dysregulation contributes to the pathogenesis of various diseases in mammals [82,83,84,85]. Over the last 10 years, autophagy (and the key mechanisms involved) have extensively been studied in relation to metabolism and mitochondrial biology in different cell and tissue types, as well as in energy homeostasis and systemic metabolism [86,87,88,89,90]. In general, basal autophagy appears to provide protein and organelle quality control by eliminating damaged cellular components whereas starvation-induced autophagy recycles intracellular components into metabolic pathways to sustain mitochondrial metabolic function and energy homeostasis [86,87]. Many excellent reviews on autophagy and metabolism have been published recently [86,87,88,89,90] and therefore, will not repeated here. Rather, we will focus on emerging evidence suggesting the importance of autophagy and its related events in cholesterol homeostasis pertaining to steroidogenesis. In steroidogenic cells, the evidence of autophagy can be traced back to 1968, when Frank and Christensen reported possible autophagic vacuoles in the interstitial cells of Guinea pig testis [91]. Subsequent studies reported the formation of autophagosomes containing mitochondria and SER [92,93]. The first report of a linkage between autophagy and testosterone production was reported in the context of late-onset hypogonadism, linking subnormal Leydig cell function with decreased autophagic activity [94]. The authors showed that the treatment of Leydig cells with an autophagy blocker, inhibited LH-stimulated StAR protein expression and decreased testosterone production, whereas treatment with an autophagy activator, enhanced LH-induced steroidogenesis. More recently, autophagy and lipophagy (i.e., the autophagic degradation of lipid droplets) have been recognized as key processes in regulating cholesterol homeostasis and its transport to the mitochondria, as well as in the maintenance of testosterone production [95,96,97]. For example, Ma et al. [95] and Khawar et al. [96] showed that autophagy and lipophagy occur in Leydig cells in response to steroidogenic stimulation, suggesting that they play a key role in cholesterol trafficking and testosterone production. Moreover, a Leydig cell-specific disruption of autophagy was found to reduce testosterone production [97].

**Figure 3:**
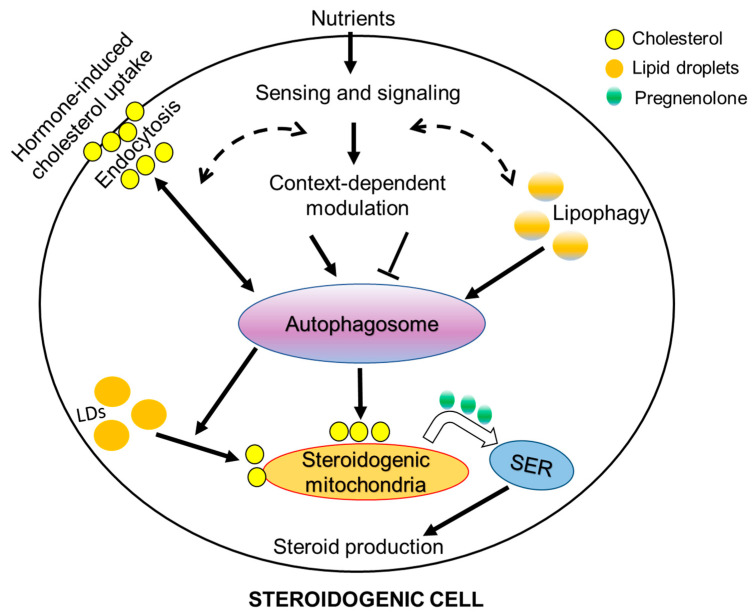
Schematic diagram depicting known (solid arrow) and potential (dashed arrow) interplay between hormone- and metabolic status-induced autophagy/lipophagy in a steroidogenic cell. It is anticipated that the interplay between these events will vary in relation to acute and chronic steroidogenesis in different steroidogenic cell types. LDs—lipid droplets; SER—smooth endoplasmic reticulum.

Lipophagy is a subtype of autophagy where the mobilization of lipids from lipid droplets are intimately linked with autophagy to deliver contents of lipid droplets to lysosomes [95]. Lipophagy has emerged as an important regulator of lipid homeostasis in different cell types. In steroidogenic cells, the utilization of cholesterol containing lipid droplets, is important for steroidogenic cells to produce different steroid hormones, which have a wide-ranging systemic effect on sexual development and immunity, inflammation, and metabolism [84]. Interestingly, the inhibition of autophagy was found to cause a decrease in lipid droplets, TGs, and cholesterol in both Leydig and adrenocortical cells [97], suggesting that autophagy plays an important role in lipid homeostasis in both steroidogenic cell types. This would imply that cell-intrinsic factors or events may regulate the dynamics of lipid droplets in steroidogenic cells (Figure 3).

Furthermore, the knockdown of Beclin-1 (a crucial autophagy gene, which is the mammalian orthologue of yeast Atg6) was found to decrease LH-stimulated StAR expression and testosterone production in mouse Leydig cells, leading to the conclusion that autophagy plays a role in the maintenance of steroidogenesis in Leydig cells. The decline in testosterone was found to be caused by a defect in cholesterol uptake in autophagy-deficient Leydig cells. Further investigations revealed that disruption of autophagic flux leads to the downregulation of the SR-BI receptors leading to insufficient cholesterol supply. Notably, in both studies, the disruption of autophagy by pharmacological or genetic approaches led to a decrease in LH-stimulated StAR expression, suggesting a link between LH-induced signaling events and autophagy in the regulation of testosterone production, which may involve cholesterol trafficking to the mitochondria, as the StAR protein plays a central role therein.

In addition to the Leydig cells and adrenocortical cells, a positive effect of autophagy has been reported in porcine granulosa cell steroidogenesis in response to FSH [98]. Mechanistically, it has been shown that FSH inhibits the activation of nuclear factor-κB, which in turn leads to the activation of Janus kinase, and consequently promotes autophagy and steroidogenesis [98], providing new insights in the regulation and function of autophagy in mammalian follicle development. Moreover, similar to Leydig cells, a disruption of autophagy by Beclin-1 deletion in ovarian luteal cells of mice was found to decrease LDs and progesterone production leading to preterm labor [97]. In aggregate, a consistent finding of the disruption of autophagy by different experimental approaches in adrenal and gonadal steroidogenic cell types suggest that autophagy and related lipophagy play a crucial role in the regulation of steroidogenesis. To the best of our knowledge, such a role of autophagy in placental steroidogenesis has not been explored yet, which warrants further investigation. In addition, it would be interesting to know whether mitophagy (selective mitochondrial autophagy) plays a role in steroidogenesis, as mitophagy has been implicated in many cell types with cell-specific mitochondrial functions. Of note, estrus cycle-related changes in steroid hormones have been implicated in selective autophagy, lipophagy and mitophagy [99]. Thus, the relationship between autophagy/lipophagy/mitophagy and steroid hormones appear to be much more complex than currently known.

In summary, these findings have shown the importance of autophagy and lipophagy in lipid regulation and steroid production. Future experiments should explore the relative importance of autophagy and select autophagy (e.g., lipophagy and mitophagy) in the basal, acute, and chronic regulation of steroid production in different steroidogenic cell types. The elucidation of these functions will be important to understand how cell-extrinsic and cell-intrinsic factors and processes coordinate to maintain various states of steroidogenesis across steroidogenic cells, which vary substantially.

Of note, most of the work on autophagy and lipophagy has been reported in relation to Leydig cell steroidogenesis [95,96,97], with only a few reports focusing on adrenocortical cells [97] and ovarian granulosa cells [84,98], and with virtually none that is focused on placental cells. Thus, the findings from one steroidogenic cell type may not be generalized to all steroidogenic cells as the physiological demands of steroidogenesis vary substantially between different steroidogenic cell types, which may necessitate cell type-specific intrinsic differences. Thus, it is important to understand the context-dependent role of autophagy/lipophagy in basal, acute, and chronic steroidogenesis in different steroidogenic cell types.

### Autophagy in Steroidogenesis—A Conserved Mechanism?

Autophagy is an evolutionarily conserved process in cell physiology, from organisms such as yeasts to mammals, which raises the question of whether autophagy’s steroidogenic role, which has been reported in many mammalian species, is also involved in other species. Recently, Texada et al. [100] showed that autophagy plays a role in the mobilization of stored precursor cholesterol and its subsequent trafficking in relation to ecdysone production in Drosophila. It was found that autophagosomes gather and transport cholesterol substrate for steroidogenesis. Thus, the results from the study by Texada et al. [100] suggest that autophagy controls the steroidogenic process by rallying LD-derived cholesterol to supply the precursor substrate for steroidogenesis, indicating a link between new evidence related to autophagy and a well-established event involved in maintaining cholesterol homeostasis in steroidogenic cells (Figure 2). The interaction of the autophagosome-mediated cholesterol-trafficking with the endosome and lysosome system supports the idea of this role, since the endocytic trafficking of cholesterol is a delivery route for steroidogenesis [101]. Thus, the cell-intrinsic events in steroidogenic cells that converts cholesterol and its intermediates into steroids might be a conserved mechanism, which requires further investigations.

## 6. Prohibitin—A Putative Novel Player in the Steroidogenic Mitochondria and Cells at Large

**Figure 4:**
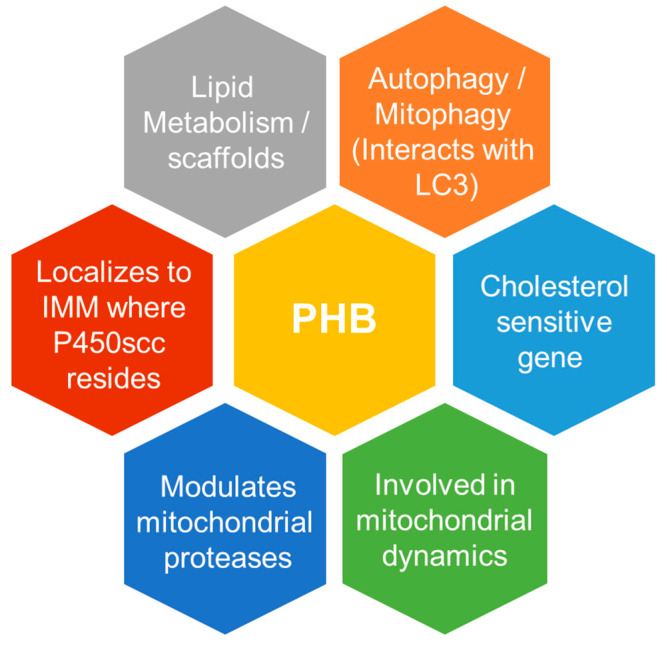
Schematic diagram depicting mitochondria, autophagy and lipid metabolism/scaffold-related known attributes of PHB that makes it an apt candidate in integrating the steroidogenic mitochondria with cholesterol mobilization and trafficking in steroidogenic cells.

Mitochondria are emerging as cellular-signaling platforms deeply integrated into diverse cellular processes. Prohibitin (PHB, also known as PHB1) is a hallmark protein of the IMM, which is involved in mitochondrial biogenesis and modulates mitochondrial dynamics [102]. PHB and its homologous protein PHB2 form large protein and lipid scaffolds (a combination of attributes that may have implications in steroidogenesis) in the IMM that are required for structural and functional integrity of the mitochondria [103] (Figure 4).

Both PHBs belong to a group of proteins family, which are thought to function as lipid and protein scaffolds in the IMM that affect the lateral distribution of the membrane lipid and protein components [104,105]. PHBs form hetero-oligomeric mega complexes composed of multiple PHB1 and PHB2 subunits [106]. In mitochondria, the PHB complex interacts with the m-AAA and other proteases, which act as a quality control enzyme with important regulatory functions in the IMM [107]. Moreover, the PHB family member protein SLP2 anchors a proteolytic hub in mitochondria containing PARL and the i-AAA protease YME1L [108,109], which are known to play a role in mitochondrial dynamics and autophagy/mitophagy. Thus, PHBs may affect mitochondrial activity in steroidogenic cells by modulating the turnover of a short-lived regulatory protein by the m-AAA protease, such as the acute regulation of StAR during steroidogenesis. In addition, PHBs may play a role in the regulation of autophagy and lipophagy because both proteins contain LC3 binding motifs and interact with each other [110], and are highly expressed in steroidogenic cells/tissues (The Human Protein Atlas).

Cholesterol serves as the metabolic precursor of all steroid hormones, and as such, steroidogenic cells and tissues can be seen as highly specialized lipid-processing cells and tissues. Since PHB has been implicated in lipid metabolism and homeostasis across species, including in mitochondrial phospholipids, and in autophagy/mitochondrial proteases, we speculate that PHB might be involved in steroid biosynthesis via lipid/cholesterol homeostasis across steroidogenic cells/tissue types. During autophagy, LC3-I is conjugated into phosphatidylethanolamine (PE) to form the LC3-PE conjugate, which is then tightly bound to the autophagosomal membranes [111]. Notably, PHB has relationship with both as it serves as a binding site for LC3 and is involved in PE synthesis.

PHB not only interacts with LC3 but is also associated with the biology of mitochondrial phospholipids, including PE [112]. It is tempting to conclude that these findings related to the autophagic regulation of factors important for cholesterol uptake and utilization in one steroidogenic cell type could be relevant for a functional interpretation across all steroidogenic cell types. However, it is likely that substantial differences between cell types are expected to exist, because the physiological demands of steroid hormones vary substantially, which are apparent in structural differences in their structure.

### The Relationship between PHB Family Proteins and Cholesterol

In addition to mitochondrial biology and autophagy, the PHB family member proteins Erlin-1 and Erlin-2 are shown to be highly enriched in the detergent-soluble, buoyant fraction of sucrose gradients in a cholesterol-dependent manner [113]. However, unlike other PHB family members (which localize to the mitochondria), these two proteins are localized to the ER. In addition to membrane localization, a common feature reported on the PHB family of proteins is that they undergo post-translational modification by palmitoylation, which is a process located in proximity of membrane targeting sequences [114]. Moreover, in a separate study [115], it was found that PHB is a cholesterol-sensitive gene, and its expression levels increase when cholesterol levels are low. In addition, the authors showed that the prohibitin gene promoter contains regulatory elements that respond to cholesterol insufficiency [115].

Moreover, the PHB family member protein MEC-2 and Podocin have both been found to bind cholesterol to regulate the activity of associated ion channels [114]. This binding requires the PHB domain, including conserved palmitoylation sites within it and a part of the N-terminal hydrophobic domain that attaches the proteins to the cytosolic side of the plasma membrane [114]. By binding to MEC-2 and Podocin, cholesterol associates with ion-channel complexes to which these proteins bind [114]. Thus, MEC-2, Podocin, and likely many other PHB-domain proteins regulate the formation and function of large protein–cholesterol supercomplexes in the plasma membrane by forming a multimeric complex among themselves, cholesterol, and different target proteins. Moreover, in mitochondria, PHB is anchored to the IMM, and forms complexes with the group of proteases known as ATPases (m-AAA), which are associated with diverse cellular activities.

Furthermore, PHB has been reported to play a role in granulosa cells [116]. However, the focus of these studies was on granulosa cell proliferation, differentiation, survival, and apoptosis rather than steroidogenesis [117,118,119] because of our existing knowledge of PHB’s context-dependent role in cell proliferation, survival, and apoptosis in different cell types. For example, Choudhury et al. [118] reported that the administration of equine chorionic gonadotropin (eCG) increases PHB expression in ovarian follicles and GC, but not in theca-interstitial cells within the pre-antral follicles. This increased expression of PHB corresponded with follicular growth and decreased after the ovulatory luteinizing hormone (LH) surge and during follicular atresia. This finding would imply that the LH surge during the ovarian cycle may negatively regulate PHB expression. Moreover, a change in the phosphorylation levels of PHB and increased trafficking to the mitochondria was observed. Notably, the PHB phosphorylation sites under these culture conditions in response to FSH and testosterone were the Tyr249, Thr258 and Tyr259 sites [118,120], which we reported in relation to insulin signaling and lipid binding/metabolism [121,122,123,124]. Thus, a possibility exists that the phosphorylation of PHB may play a role in steroidogenic cells in response to hormones and growth factors (e.g., trophic hormones, insulin, IGF and EGF), which are known to stimulate steroidogenesis.

Most of the arguments on the putative role of PHB in steroidogenesis in this section are hypothetical and are based on previous research findings on PHB in mitochondrial biology and lipid metabolism. Almost none of PHB’s mitochondrial and lipid metabolism attributes has been reported in relation to steroidogenesis. However, new unpublished findings from our laboratory are suggestive of PHB playing an important role in autophagy/lipophagy, cholesterol homeostasis and in mitochondrial dynamics in steroidogenic cells. It is our hope that others will see many research opportunities here, and that they will carry out studies that test these ideas.

## 7. Outstanding Questions and Future Research Directions

The emergence of the role of mitochondria from the site of initiation of steroidogenesis to the regulator of cholesterol mobilization, trafficking, and homeostasis to support the body’s physiological levels of steroid hormone production have provided new insights and created exciting future research directions. One such example is the putative role that PHB plays in integrating various aspects of steroidogenic mitochondria, because of many fitting attributes it possesses related to mitochondrial biology and lipid metabolism (Figure 4). However, a number of fundamental questions related to our current understanding of steroidogenesis remain unanswered (Appendix A). Emerging pieces of knowledge about steroidogenesis have created opportunities to use a fresh approach to understand these underlying questions. It is expected that unraveling the molecular understanding of factors that finely tune steroid hormone production and avoid hormone insufficiency or excess may lead to the development of new therapeutic opportunities for the treatment of various diseases associated with their dysregulation.

## Appendix A. Outstanding Questions

Q1. Why is steroidogenesis compartmentalized to membranes that are poor in cholesterol content (i.e., IMM and SER), but not in the PM, which is rich in cholesterol?
Q2. Why are steroidogenic enzymes membrane bound, unlike many other metabolic enzymes?
Q3. Do steroidogenic enzymes that are located in the SER play a role in SER functions like steroidogenic enzymes present in the IMM in mitochondrial function?
Q4. What is the relative importance of autophagy and lipophagy in fulfilling steroidogenic cholesterol requirements under situations of cholesterol sufficiency and insufficiency?
Q5. Why is the cell’s StAR level acutely regulated in response to pituitary tropic hormones? What is the role of mitochondria in the regulation of StAR turnover?
Q6. Does PHB play a role in the functional coupling of StAR and P450scc, acute regulation of StAR and in the localization of steroidogenic enzymes in the IMM?
Q7. How do autophagy, lipophagy and mitochondrial dynamics operate under cholesterol-deficient and -sufficient states?
Q8. Does mitophagy play a role in steroidogenesis?
Q9. What is the relative importance of autophagy and lipophagy in basal, acute, and chronic steroidogenesis in different steroidogenic cell types?
Q10. What are the factors and mechanisms involved in cholesterol transport to the IMM?
Q11. Are syncytiotrophoblast mitochondria different from the mitochondria of the adrenal cortex and gonadal cells?
Q12. Do the autophagy and lipophagy processes that have been reported to play roles in steroidogenesis in gonadal cells also take part in placental steroidogenesis?
